# Reprocessable Photodeformable Azobenzene Polymers

**DOI:** 10.3390/molecules26154455

**Published:** 2021-07-23

**Authors:** Huiqi Zhang

**Affiliations:** State Key Laboratory of Medicinal Chemical Biology, Key Laboratory of Functional Polymer Materials (Ministry of Education), Collaborative Innovation Center of Chemical Science and Engineering (Tianjin), College of Chemistry, Nankai University, Tianjin 300071, China; zhanghuiqi@nankai.edu.cn

**Keywords:** azobenzene polymers, photodeformable, reprocessable, dynamic crosslinking networks

## Abstract

Photodeformable azobenzene (azo) polymers are a class of smart polymers that can efficiently convert light energy into mechanical power, holding great promise in various photoactuating applications. They are typically of crosslinked polymer networks with highly oriented azo mesogens embedded inside. Upon exposure to the light of appropriate wavelength, they experience dramatic order parameter change following the configuration change of the azo units. This could result in the generation and accumulation of the gradient microscopic photomechanical force in the crosslinked polymer networks, thus leading to their macroscopic deformation. So far, a great number of photodeformable azo polymers have been developed, including some unoriented ones showing photodeformation based on different mechanisms. Among them, photodeformable azo polymers with dynamic crosslinking networks (and some uncrosslinked ones) have aroused particular interest recently because of their obvious advantages over those with stable chemical crosslinking structures such as high recyclability and reprocessability. In this paper, I provide a detailed overview of the recent progress in such reprocessable photodeformable polymers. In addition, some challenges and perspectives are also presented.

## 1. Introduction

Photodeformable azobenzene (azo) polymers are a class of smart functional polymers that can convert light energy into macroscopic displacement or deformation (i.e., photomechanical effects) [1,2,3,4,5,6,7,8,9,10,11,12,13,14,15]. As a photoswitchable chromophore, azo unit can reversibly change the conformation between two structural configurations (i.e., *trans* and *cis* isomers) with high quantum efficiency and fatigue resistance [16,17]. In particular, the photoisomerization of an azo unit from the rod-like *trans*-form to the bent *cis*-form can lead to its large size (or length) alteration from 0.9 to 0.55 nm, which can lead to dramatic changes in the alignment order of azo mesogens and the conformation of their surrounding polymer chains, thus resulting in the generation of microscopic force inside azo polymers and their photomechanical effects. They can show various macroscopic photodeformation behaviors under the irradiation of appropriate light such as contraction, bending, twisting, oscillation, rotation and translational motion. They have shown great potential in a wide range of actuating applications such as soft robots, artificial muscles, and microfluidic devices.

Although some photodeformable azo polymers were reported as early as 1960–1980s, they only exhibited very small photoinduced shape changes (typically ≤4%) [18,19,20]. It is Finkelmann and coworkers who developed the first series of photodeformable azo polymers with remarkable shape change in 2001 [21]. They successfully prepared the monodomain nematic liquid crystalline (LC) elastomers (LCEs) by introducing both photoresponsive azo mesogens and photoinert mesogens into the crosslinked polysiloxane networks through the combined use of Pt-catalyzed addition reaction between C=C double bond and Si-H unit and a “two-step crosslinking” strategy. Such uniaxially oriented monodomain nematic LCEs showed large and reversible UV/visible light-induced contraction/expansion (up to around 20%) along the mesogen alignment direction due to the UV-induced reduction and visible light-induced restoration of the nematic order and the simultaneous conformation change of the polymer chains. Later on, Ikeda and coworkers disclosed that a free-standing polydomain film based on the crosslinked azo polymer LC network (LCN) prepared by the thermal copolymerization of a monoacrylate azo monomer and a diacrylate azo crosslinker could repeatedly and precisely bend along any chosen direction by using the linearly polarized light [22]. This reversible photodeformation behavior was induced by the subtle reduction in microscopic ordering of the LC domains and volume contraction at the film surface, mainly because of the selective absorption of the polarized light by the azo moieties that are aligned along the direction of light polarization in the specific domains only at the film surface layer (owing to the strong light absorption by *trans*-azo moieties at about 360 nm) and their *trans*–*cis* isomerization. Subsequently, Ikeda’s group also developed glassy monodomain LCN films by polymerizing a monoacrylate azo monomer and a diacrylate azo monomer in a LC cell, which proved to show obvious photoinduced bending/unbending behaviors along the azo mesogen alignment direction under the irradiation of nonpolarized UV/visible light [23]. These milestone works have significantly spurred the interests of scientists from different disciplines in these novel stimuli-responsive smart materials and largely promoted the development of this research field.

So far, a great number of photodeformable azo polymers with various chemical structures have been developed via different synthetic strategies [1,2,3,4,5,6,7,8,9,10,11,12,13,14,15]. They are typically prepared by first orientating the azo mesogens (or together with other mesogens) through mechanical stretching or surface alignment and the subsequent locking of the ordered structures through various crosslinking reactions. Recent years have witnessed significant progress made in the molecular design, development of orientation methods of the mesogenic units and various deformation formats, miniaturization, and performance improvement of the azo polymer photoactuators. According to the location of the azo units in the polymer chains, photodeformable azo polymers can be mainly divided into side-chain and main-chain types. Many of their structural parameters including polymer backbone structure, azo content and its location, orientation degree and direction of the mesogenic units, crosslinking degrees, and sample sizes (e.g., the thickness of the films and diameters of the fibers) have proven to show significant influence on their photomechanical effects. In particular, the initial orientation degrees of the mesogenic units and their coupling ability with polymer chains in the crosslinked networks have proven to be the paramount influencing factor [2,3]. The photodeformable azo polymers have proven to show enhanced photodeformation behaviors with an increase in their initial orientation degrees of the mesogenic units [23]. In addition, an increase in the coupling ability between the mesogenic groups and polymer chains can lead to a higher degree of the anisotropy of the polymer chain conformation in the obtained orientation systems, which can lead to their more significant photomechanical effects [3]. In comparison with photodeformable side-chain azo polymers, the main-chain ones could possess much better photomechanical properties because of their stronger orientation coupling between the (azo) mesogens and polymer chains [2,3,8,24]. It is also worth noting here that the change in the location and orientation of azo chromophores in the polymer chains (main- or side-chains) can change the photodeformation direction of azo polymers, as was shown in experiments [25,26] and in theoretical works [27,28].

**Figure 1:**
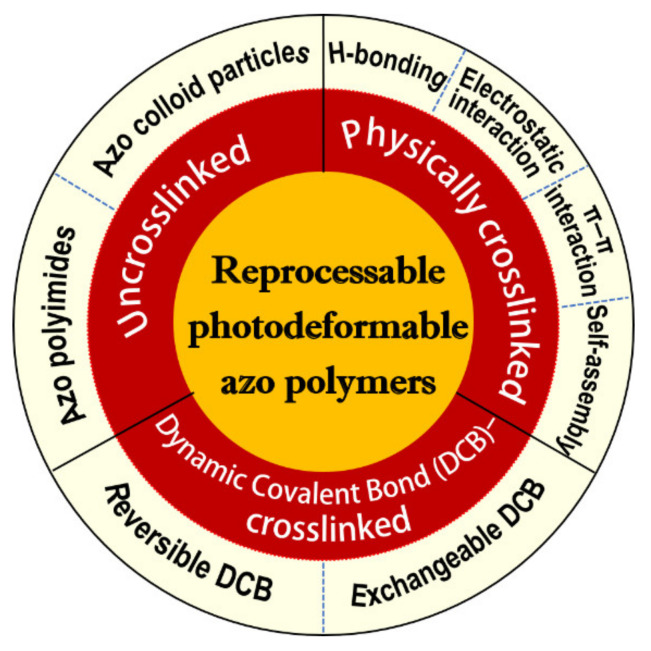
Schematic illustration for the outline of this review paper.

It has also been well demonstrated that the presence of an appropriate crosslinking degree in the photodeformable azo polymer actuators is essential for them to transport the mechanical force generated by the photoisomerization of azo mesogens to the whole system and induce the reversible macroscopic motion [4,29,30,31,32]. Among all the presently developed photodeformable azo polymers, most of them have stable chemical crosslinking structures. Although they could exhibit good photomechanical and mechanical properties, they have some obvious drawbacks such as the typical complicated crosslinking processes, low flexibility in fabrication, and less freedom for further processing and recycling. To address these issues, a number of reprocessable photodeformable azo polymers have been developed on the basis of different design strategies [29,31,33,34,35,36,37,38,39,40,41,42,43,44,45,46,47,48,49,50,51,52,53,54,55,56,57,58,59,60,61,62,63,64,65,66,67,68,69,70,71,72,73,74,75,76,77,78,79,80,81,82,83,84,85,86]. They can be mainly classified into two types, i.e., the physically crosslinked ones with dynamic non-covalent bond-involved networks [29,31,33,34,35,36,37,38,39,40,41,42,43,44,45,46,47,48,49,50,51,52,53,54,55,56,57,58,59,60] and chemically crosslinked ones with dynamic covalent bond (DCB)-involved networks [61,62,63,64,65,66,67,68,69,70,71]. In addition, some uncrosslinked photodeformable azo polymers have also been reported and constitute one special type of reprocessable photodeformable azo polymers, whose photodeformation behaviors are mainly induced by the selective reorientation of the azo moieties (become perpendicular to the polarization direction of the polarized light) under the irradiation of either polarized blue light [72,73,74,75,76,77] or interfering polarized light [78,79,80,81,82,83,84,85,86] (note that the light sources used for the photodeformation studies in the following parts are the nonpolarized light unless otherwise stated). In the following section, I present a detailed overview on these versatile reprocessable photodeformable azo polymers. In this context, it is also noteworthy that another type of reprocessable photodeformable linear azo polymers that can show large surface morphology change (i.e., surface relief gratings) upon exposure to the interfering polarized light is not included in this paper. The readers are recommended to refer to some review papers related to this topic [87,88,89]. The outline of this review is shown in Figure 1.

## 2. Reprocessable Physically Crosslinked Photodeformable Azo Polymers

The reprocessable physically crosslinked photodeformable azo polymers are photodeformable polymers with crosslinked networks that are formed through various supramolecular non-covalent interactions including hydrogen bonding (H-bonding) [29,31,33,34,35,36,37,38,39,40,41,42,43,44,45,46,47], electrostatic interaction [48,49], π-π interaction [50,51], and other self-assembly-induced interactions [52,53,54,55,56,57,58,59,60]. To date, their preparation methods can be mainly divided into one-step and two-step approaches. In the former case, photodeformable azo polymers with non-covalent interacting groups in their side-chains or main-chains are directly prepared in one pot via different polymerization methods, which can form physically crosslinked networks themselves [29,31,35,36,37,43,44,45,46,47,50,51,52,53,54,55,56,57,58,59,60]. In the latter case, physically crosslinked photodeformable azo polymers are prepared via first the synthesis of azo polymers or acquiring some commercially available polymers (without azo groups) bearing non-covalent interacting groups (A groups) and their subsequent mixing with certain azo crosslinkers bearing dual or multiple non-covalent interacting groups (B groups) that can form supramolecular interactions with A groups [33,34,38,39,40,41,42,48,49]. Since their preparation procedures do not involve the typically complicated chemical crosslinking step, they normally can be more easily fabricated than the chemically crosslinked photodeformable polymers. In particular, owing to the dynamic nature of the non-covalent interactions, these photoactuators can be recycled and further reprocessed after their use or even have self-healing ability, which make them highly promising in real-world applications. Below, I present a detailed overview of the reprocessable physically crosslinked azo polymers according to their utilized different non-covalent interactions.

### 2.1. Hydrogen Bonding (H-bonding) Interactions

Hydrogen bonds (H-bonds) are readily formed between a donor with an available acidic hydrogen atom and an acceptor carrying a nonbonding lone pair of electrons. They are highly selective and directional and can show environmental stimulus-responsivity owing to the dependence of their strength on the solvent and temperature. Therefore, recent years have witnessed tremendous interest in the application of H-bonds for the construction of various supramolecular polymer systems including supramolecular azo polymers [90,91,92,93]. To date, a series of supramolecular H-bonded physically crosslinked photodeformable azo polymers have also been developed. They can be mainly divided into side-chain and main-chain types (on the basis of the location of the azo units) as shown below.

#### 2.1.1. H-Bond-Crosslinked Photodeformable Side-Chain Azo Polymers

**Figure 2:**
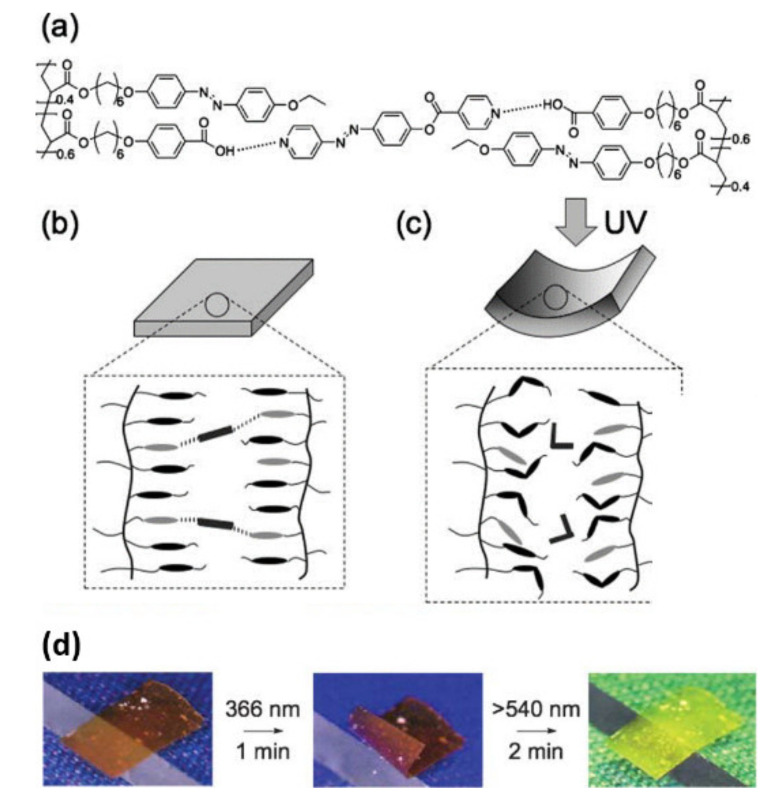
(**a**–**c**) Plausible mechanism of bending in the H-bonded crosslinked LCP film of an azo copolymer and an azo crosslinker: (**a**) network structure of the H-bonded crosslinked LCP film consisting of the azo copolymer and the azo crosslinker; (**b**,**c**) schematic illustration of molecular alignment in the H-bonded crosslinked LCP film before (**b**) and after (**c**) irradiation with UV light. (**d**) Photoresponsive behavior of the H-bonded crosslinked LCP film. Size of the film: 2 mm × 3 mm × 20 μm. UV light intensity: 18 mW cm^−2^; visible light intensity: 21 mW cm^−2^. Reprinted with permission from Reference [33]. Copyright 2008 Royal Society of Chemistry.

In 2008, Ikeda and coworkers reported the first example of a supramolecular photomechanical system based on the H-bonded physically crosslinked azo LC polymer (LCP) film [33]. A side-chain polyacrylate with pendant benzoic acid units and ethoxyl group-terminated azo groups (PAAC) and an azo crosslinker with a pyridine unit at its both ends (PEAP) were used for such a purpose (Figure 2a). A free-standing physically crosslinked film with preferentially aligned azo mesogens along the film surface was fabricated by putting a melt mixture of PAAC and PEAP between two NaCl plates with rubbing treatment (Figure 2b). Under the irradiation of UV/visible light, the azo polymer film showed photoinduced bending and unbending (Figure 2c,d). In addition, this physically crosslinked LCP film could be recycled and reconstructed through its dissolution in tetrahydrofuran (THF), reprecipitation of its THF solution into diethyl ether, and finally being sandwiched between NaCl plates with rubbing surfaces. The reconstructed azo polymer film also exhibited reversible photoinduced bending. The macroscopic bending of this physically crosslinked film was attributed to the decrease in the alignment order of H-bonded LC structures during the *trans* to *cis* photoisomerization of the azo crosslinker (as also demonstrated by the theoretical consideration of the photoisomerization process in LCPs [28]). This work opens up a highly versatile way toward developing advanced light-driven actuators using supramolecular assemblies of azo polymers. In a following work from the same group, the surface-modified single-walled carbon nanotubes (SWNTs) (with carboxylic acids) were introduced to enhance the mechanical stability of such supramolecular H-bonded polymer actuators [34]. The polymer fibers prepared from a mixture of PAAC, PEAP, and the modified SWNTs (0.1%) via the melt spinning method could show rapid and reversible photoinduced bending and unbending. They also exhibited largely enhanced mechanical strength and photoinduced stress. Particularly, PAAC and PEAP could also be recovered by solvent extraction, reused, and recycled. It is worth mentioning here that enhancing the photoinduced stress of the azo photoactuators is highly important for their practical applications. Rather high light-induced mechanical stress (0.4–1.2 GPa) has been experimentally achieved [94,95,96] (and also confirmed by theoretical calculations [97]), which could dissociate covalent C-C bonds [94] and break metallic layers on the surface of glassy azo polymers [95].

Yu and coworkers reported a photodriven swing actuator composed of a commercially available polyimide (Kapton) substrate layer (with a high elastic modulus) and a photoresponsive LCP layer [35]. The photoresponsive LCPs were prepared by the free radical copolymerization of one benzenecarboxylic acid-containing methacrylate monomer and one methacrylate azo monomer in different molar ratios. The thermally annealed bilayer film displayed chaotic swing under the continuous irradiation of the actinic light. The *trans* to *cis* azo photoisomerization rate of the LCPs and the swing amplitude of the bilayer film were largely enhanced by the introduction of H-bond-forming benzenecarboxylic acid unit into the copolymers and the existence of supramolecular H-bonding in LCP films, respectively. Moreover, the presence of the supramolecular H-bonded physical crosslinking in the LCP layer could not only enhance the driving force for photomechanical deformation, but also improve the elastic modulus of the photoactive layer and modulate the swing behavior of the bilayer strip. More importantly, the formation of H-bond in the form of acidic dimers has a spatial confinement effect, extending the timescale of photodriven swing. The photomechanical self-vibration of the bilayer film was attributed to the combined azo photoisomerization and local photosoftening effect of LCPs.

Self-healing polymers have garnered great interest because of their increased lifetime, safety, and sustainability [98,99,100]. Recently, some H-bonded physically crosslinked (photodeformable) side-chain azo polymers with self-healing properties were developed by Zhang and coworkers [36,37]. They first prepared a series of rod-coil block copolymers consisting of a random side-chain azo copolymer (with a polymethacrylate backbone and both pendant butyl-terminated azo mesogens and hexyl units) as the rod block and polymethacrylate with pendant amide groups as the coil block via the sequential reversible addition-fragmentation chain transfer (RAFT) polymerization and studied their self-healing properties and phase structures [36]. These rod-coil block copolymers could show rubber-like features at room temperature and self-healing properties under the mild heating condition (50 °C). The H-bonding that formed between the amide groups on the polymer chains was found to play an important role in the microphase separation and morphology of the polymers. The incorporation of such supramolecular H-bonding interactions into these polymers could suppress the microphase separation but enhance their LC order. However, the photodeformation results of these azo polymers were not presented.

2-Ureido-4[1H]-pyrimidinone (UPy) unit has been widely used for the construction of self-healing and malleable polymers with high mechanical properties [101,102], mainly because it has strong dimerization capability through forming quadruple H-bonding with an association constant of 6 × 10^7^ M^−1^ (in deuterated chloroform) [90]. This quadruple H-bonding-forming unit was also introduced into the azo polymers to prepare reprocessable photodeformable azo polymers with self-healing ability by Zhang and coworkers [37]. Several side-chain azo copolymers bearing different contents of pendant alkyl-terminated azo mesogen, UPy, and butyl acrylate unit (BA, functioning as the internal plasticizer to reduce the glass transition temperature (*T*_g_) of the polymers) were prepared via the traditional free radical copolymerization method. They showed enhanced mechanical strength and self-healing properties owing to the formation of multivalent H-bonds between their UPy units. An azo copolymer with an optimal composition (i.e., 57.7% Azo, 32.6% BA, and 9.7% UPy) was used to prepare uniaxially oriented fibers (with azo mesogens aligned along the fiber axes) and well-oriented free-standing films (with azo mesogen alignment homeotropic to the film surfaces) by using the melt spinning method and a special melt pressing approach, respectively. The melt pressing approach involves first sandwiching the azo copolymer between two pieces of glass coated with sodium polyacrylate (PAANa), pressing on the glass plates to compress the melt spread out into a thin film, annealing the thin film at 70 °C for 3 h, and then immersing the sandwiched film into water to obtain the free-standing film by dissolving PAANa. Both the azo polymer fiber and film could show photoinduced bending and unbending behaviors. While the azo polymer fiber bent toward the incident UV light, the azo polymer film bent away from the UV source, which could be attributed to their different orientation mechanism [103]. Moreover, the azo polymer samples also exhibited good self-healing properties at 40 °C, which is highly beneficial to enhance the life longevity of these photoactuators.

Some reprocessable H-bond-crosslinked photodeformable azo polymers have also been developed through simply mixing small azo compounds with commercially available polymers [38,39,40,41,42], following a similar strategy widely used for the preparation of supramolecular azo polymers [92]. Feng and coworkers reported the controllable and stable deformation of a self-healable photoresponsive supramolecular H-bonding-assisted assembly for an optically actuated manipulator arm [38]. A supramolecular H-bonded free-standing film with stabilized *cis-*azo isomer was fabricated by first casting a mixed solution of 3,3′,5,5′-azobenzenetetracarboxylic acid (t-Azo) and polyacrylic acid (PAA) grafted with UPy (PAA-u) in DMF onto a quartz glass, irradiating the solution with UV light, and finally peeling off the dried film from the glass. In comparison with the typical photodeformable azo polymers, the uniqueness of this work is to improve the deformation stability of the physically crosslinked azo polymers by inducing the deformation through *cis*-to-*trans* azo isomerization under the green light irradiation. The deformation of the PAA-u/t-Azo film with *cis*-rich states not only showed high stability but also could recover the shape under UV irradiation. Based on such reversible photodeformation, an optically actuated manipulator arm was designed, which allowed cycling, grabbing, and releasing an object upon optimizing the “finger” shape of the “hand”. Moreover, this manipulator arm also showed a fast self-healing performance under green-light irradiation. Zhao and coworkers developed some photodeformable azo polymers through the H-bond-assisted self-assembly of 4,4′-dihydroxyazobenzene and chitosan [39]. The physically crosslinked polymer films were readily fabricated through mixing the solution of chitosan in 2% acetic acid and the solution of azo compound in methanol and allowing the evaporation of the solvents. They could show obvious photoinduced bending and lift a weight which is up to 200 times of that of the actuator under 355 nm light irradiation. Yu and coworkers fabricated some photoresponsive actuator films from polymer-dispersed LC (PDLC)/graphene oxide (GO) nanocomposites through first preparing a homogeneous suspended solution of a polymer [polyvinyl alcohol (PVA) [40] or polyurethane (PU) [41]], organic LC compounds (including both a photoinert LC compound and an azo compound), and GO and then casting it onto the clean glass slide, which could show obvious NIR-vis-UV light-induced deformation based on the photothermal and photochemical phase transition of the LC microdomains in the composite films [40,41]. Wu and coworkers reported a solar actuator based on H-bonded azo polymers for electricity generation, which was fabricated by introducing 2,2′,6,6′-tetrafluoro-4,4′-diacetamidoazobenzene (F-Azo) into agarose (AG) [42]. The resulting physically crosslinked F-Azo-doped AG (F-Azo@AG) films bent under sunlight irradiation, and such photoinduced bending could transduce the sunlight into electricity when the F-Azo@AG film was attached to a piezoelectric transducer.

All the above-described H-bond-crosslinked supramolecular azo polymers are side-chain type. As mentioned in the Introduction section, main-chain azo polymer photoactuators could show better photodeformation behaviors and higher mechanical strength than the side-chain ones because of the stronger coupling between the azo mesogens and polymer backbones (resulting in higher orientation degrees) and structural characteristics. Therefore, the development of physically crosslinked main-chain azo polymer photoactuators is of significant importance for achieving advanced actuators.

#### 2.1.2. H-Bond-Crosslinked Photodeformable Main-Chain Azo Polymers

**Figure 3:**
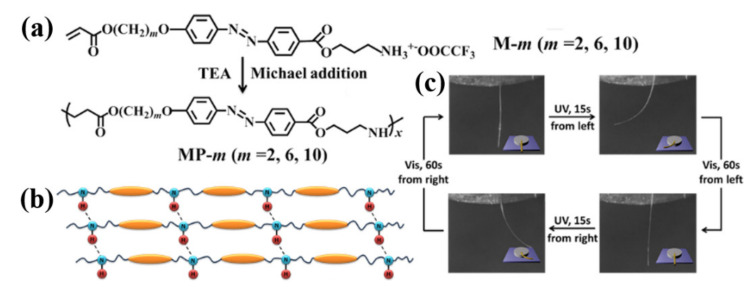
(**a**) Chemical structure and synthetic route of the main-chain azo LCP with both ester and secondary amino units in its backbone. (**b**) Supramolecular H-bonded physical crosslinking formed between main-chain azo polymer chains. (**c**) Photographs of the photodeformation behaviors of a main-chain azo polymer fiber fabricated by using melt spinning method. The fiber reversibly bends upon irradiation with UV and visible light. Reprinted with permission from Reference [29]. Copyright 2013 American Chemical Society.

In 2013, our group developed a versatile approach for preparing physically crosslinked photodeformable main-chain azo LCPs with both ester and secondary amino units in their backbones (Figure 3a,b) [29]. It involves first the synthesis of a series of acrylate-type azo monomers bearing an amino terminal group and their subsequent Michael addition polymerization under the mild condition (Figure 3a). These main-chain azo polymers showed high thermal stability, relatively low *T*_g_ (down to 30 °C as determined by DSC), a broad temperature range of smectic C mesophases, and reversible photoresponsivity. Their uniaxially oriented fibers with a high alignment order of azo mesogens along the fiber axes and easily tunable diameters were readily fabricated by using the simple melt spinning method. They showed rapid and reversible photoinduced deformation even at close to ambient temperature (Figure 3c). They could produce photoinduced stress around 240 kPa under the UV light irradiation (150 mW/cm^2^), which is close to the stress generated by the chemically crosslinked azo LCP films [104] and fibers [105] as well as that of the human muscles (around 300 kPa) [104,105]. In addition, the polymer fiber achieved a tensile strength of 44 MPa and a tensile modulus of 5.8 GPa, which are about 2.8 and 14.5 times as large as the tensile strength (i.e., 16 MPa) and modulus (i.e., 400 MPa) of a chemically crosslinked azo LCP film, respectively [106]. The H-bonds that formed among the secondary amino groups proved to play a decisive role in the photomobility of the uniaxially oriented fibers. These findings represent the first successful example of the supramolecular photomechanical system based on the main-chain LCPs with azo mesogens in their backbones that can show good mechanical properties, fast and reversible photoinduced bending/unbending and large photoinduced stress at close to ambient temperature, and excellent photomobile fatigue resistance. The highly appealing properties of such supramolecular H-bonded photomechanical system, such as flexible preparation, high reconstruction and recycle ability, and good mechanical and photomechanical properties, make it very promising in various photoactuating applications.

Following the above work, we also prepared a physically crosslinked photodeformable main-chain azo poly(ester-amide) (PEA) with both ester and amide groups in its backbone via first the synthesis of an acrylate-type azo monomer with an *N*-hydroxysuccinimide carboxylate end-group and its subsequent Michael addition/amidation cooperative polymerization with cysteamine under mild conditions [31]. The obtained azo polymer exhibited high thermal stability, a low *T*_g_ (43 °C), a broad range of smectic LC phases, and reversible photoresponsivity. Its uniaxially oriented supramolecular H-bonded fibers fabricated via the melt spinning method showed much higher mechanical strength than many physically and chemically crosslinked photomobile side-chain LCP systems. In addition, they exhibited photoinduced bending/unbending with high fatigue resistance as well as more rapid photodeformation rate than both the above-described physically crosslinked main-chain LCPs (with both ester and secondary amino groups in the backbones) and some typical chemically crosslinked side-chain LCP-based fibers even at ambient temperature. The H-bonding-induced physically crosslinked network (by the amide groups in the PEA) also played a decisive role in the photomobility of PEA fibers. Very recently, we further demonstrated the successful synthesis of a new main-chain crystalline azo polymer with both ester and secondary amino groups in its backbone and photodeformation behaviors of its supramolecular hydrogen-bonded fibers [43]. This azo polymer was prepared via first the synthesis of a diacrylate-type azo monomer and its subsequent Michael addition copolymerization with *trans*-1,4-cyclohexanediamine under the mild reaction condition. It proved to have good thermal stability, low *T*_g_, broad crystalline phase temperature range, and obvious photoresponsivity. Its uniaxially oriented physically crosslinked supramolecular H-bonded fibers with good mechanical properties were readily fabricated by using the melt spinning method, and they could exhibit “reversible” photoinduced bending and good fatigue resistance under the irradiation of the same UV light alone. Such photodeformable main-chain azo polymer fibers can also be recycled for reuse due to their physical crosslinking characteristics.

Later on, White and Tan’s group also reported the photomechanical response of an amorphous azo-functionalized polyamide (azoPA) (with its *T*_g_ at 220 °C (determined by DSC) and elastic storage modulus (*E*’) being ca. 2.5 GPa at 25 °C (by DMA)) [44]. AzoPA was prepared via the polycondensation of 4,4′-oxydibenzoyl chloride and 2,2-bis{4-[4-(4-aminophenyldiazenyl)phenoxy]phenyl}propane with pyridine as the HCl scavenger. Upon exposure to the polarized blue laser light (*λ* = 445 nm) (with its polarization direction parallel to the long axis of the azoPA cantilever), the unoriented isotropic cantilever of azoPA could show large photoinduced deflection (bending) toward the incident light because of the *trans*–*cis*-*trans* photoisomerization of the azo units and their reorientation perpendicular to the polarization direction of the blue light. The indirect influence of light on the rupture of intermolecular H-bonding and the concurrent reduction in stiffness were considered to be responsible for the enhanced photomechanical response of azoPA. In addition, the reformation of H-bonding in azoPA after turning off the light could lead to the full recovery of the photomechanical properties (including the unbending and restoration of the modulus of the azoPA cantilever) after relaxation in the dark.

Some other physically crosslinked photodeformable main-chain azo polymers containing H-bond-forming units in their backbones have also been developed in the past few years, including PU [45], polyurea [46], and poly(urethane-urea) [47]. Zhuo and Chen’s groups reported the synthesis of an azo PU and its use as the staging-responsive shape memory polymers (SR-SMPs) [45]. It was prepared via the polymerization of *N*-methyldiethanolamine (MDEA), 4,4-azodibenzoic acid (Azoa), and hexamethylene diisocyanate (HDI). The uniaxially oriented azo PU showed rapid photoinduced curl deformation under UV irradiation, whose shape could be kept unchanged at room temperature even upon its exposure to visible light (probably owing to its increased H-bonding interactions between the molecular chains and thus enhanced *T*_g_ after the UV irradiation) but recovered its original shape upon heating. The use of a linear photoresponsive polyurea with bridged azo moieties in its backbone (PbAzo) as visible-light-driven actuator and reversible photopatterning was reported by Zhang and coworkers [46]. PbAzo was prepared via the polyaddition reaction between HDI and *cis*-3,3′-diamino ethylene-bridged azo compound (bAzo) (its weight-average molecular weight determined by GPC, *M*_w,GPC_ = 5 kg/mol). bAzo has a thermally stable *cis*-isomer form and a metastable *trans*-isomer form at room temperature because of the ring strain [107]. It can exhibit *cis*-to-*trans* and *trans*-to-*cis* photoisomerization under the irradiation of 405 nm blue light and 532 nm green light, respectively. The *cis* to *trans* transformation of bAzo led to the amorphous-to-crystalline transition and yellow-to-red color change of PbAzo. Upon exposure to 405 nm blue light, the polyurea film rapidly bent away from the light source, and then recovered to its initial state with no attenuation under irradiation with 532 nm green light. In addition, photopatterning in the PbAzo film was also realized, and the formed patterns could be reversibly written or erased alternatively by 405 nm blue light and 532 nm green light or heating. This azo polyurea shows some interesting photoresponsive properties different from photoresponsive polymers with planar azo moieties, but its low thermal stability (decomposition starts above 90 °C) might limit its applications in the photoactuating field.

Recently, Shepherd and coworkers reported the synthesis of several main-chain azo poly (urethane-urea)s showing photodeformation behaviors and self-healing properties [47]. These azo polymers were prepared via a two-stage reaction including first the synthesis of a PU prepolymer through the reaction between toluene-2,4-diisocyanate (TDI) and poly (propylene glycol) (PPG, *M*_n_ = 2000) with a molar ratio of 10:6 and its subsequent chain-extension with different molar ratios of 4,4′-methylenedianiline (MDA) and 4,4′-diaminoazobenzene (DAB). Three amorphous azo poly (urethane-urea)s with varying ratios of PPG:MDA:DAB (6:0:4, 6:1:3, 6:2:2) and similar molecular weights (*M*_w,GPC_ = 27,700–33,200 g/mol) were obtained and their azo content–property relationship was studied. These thermoplastic elastomers showed good self-healing properties owing to their presence of two kinds of dynamic non-covalent bonds. The uniaxially oriented films (prepared via the uniaxial stretching of the unstretched films to 300% strain at 60 °C) showed photoinduced bending toward the UV source and visible light-induced unbending, but the photoinduced mechanical stress, photomobile rate, and energy conversion efficiency were relatively low. A light-driven soft-robotic gripper was also fabricated by taking advantage of the self-healing and photodeformation properties of the actuating film.

### 2.2. Electrostatic Interaction

**Figure 4:**
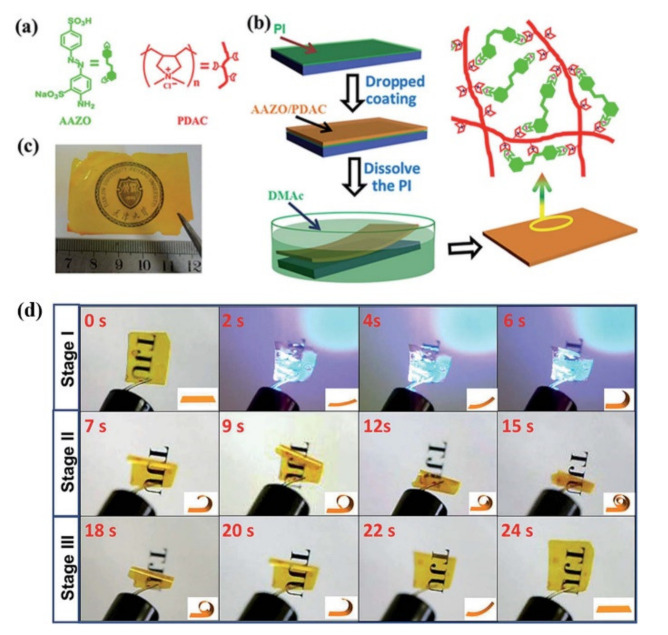
(**a**) Chemical structures of AAZO and PDAC. (**b**) Schematic illustration of the preparation and structures of the AAZO/PDAC film. (**c**) Optical image of a free-standing AAZO/PDAC film with an area of 5 cm × 3.2 cm. (**d**) Photographic frames of the light-driven deformation and shape recovery of the AAZO/PDAC film. The film (10 mm × 15 mm) was held vertically with one side clamped. The UV light (30 mW cm^−^^2^) is switched on in stage I and switched off in stage II and stage III. The inset of each photograph is a schematic illustration of the film. Reprinted with permission from Reference [48]. Copyright 2015 Royal Society of Chemistry.

The electrostatic interaction involves ions, dipoles, and induced dipoles. Recent years have witnessed considerable interest in their application in the design of various advanced functional supramolecular polymers [92,93]. It has also been successfully utilized to construct physically crosslinked azo polymer photoactuators by Feng and coworkers [48,49]. The first such supramolecular crosslinked azo polymer photoactuator was prepared by the electrostatic interaction between a photochromic azo compound with disulfonic groups (AAzo) and a cationic polyelectrolyte (PDAC) (Figure 4a–c) [48]. Under the optimal weight ratio of AAzo and PDAC (1:4), the unoriented supramolecularly self-assembled azo polymer film exhibited a large deformation under light illumination (Stage I) and continue to deform when the light was off (Stage II), along with a spontaneous shape recovery (Stage III) (Figure 4d). This photomechanical deformation demonstrated that the crosslinking by electrostatic interaction is able to convert the microscopic motion of the azo moieties into a macroscopic change of the film. It was postulated to be caused by the different rates and degrees of structural transformation of AAZO/PDAC film between the front (facing UV light) and back side with the segmental motion of polymers. Moreover, the AAZO/PDAC film also exhibited an excellent cycling performance of the photodeformation and recovery, and the rolled-up shape (large deformation) was easily fixed through soaking in DMF by disturbing the electrostatic interaction.

Feng and coworkers also constructed a multi-stimuli-responsive azo polymer actuators by self-assembling a tetrafunctional azo monomer (i.e., 3,3′,5,5′-azobenzenetetracarboxylic acid (H4abtc)) and the cationic polyelectrolyte PDAC through the electrostatic interaction [49]. A free-standing unoriented H4abtc/PDAC film was prepared by using an optimized drop-coating technique. Since the structural transformations of H4abtc can be induced by light, mechanical force, and heat, and its asymmetric structural transformations on either side of the film can generate asymmetric contraction/stretching forces, the obtained H4abtc/PDAC film was able to undergo controllable, reversible, and repeatable bending/unbending movements upon treatment with light, humidity, or temperature. Fast rates of shape recovery were achieved for the film upon exposure to gently flowing humid nitrogen (under the irradiation of visible light).

### 2.3. π–π Interaction

**Figure 5:**
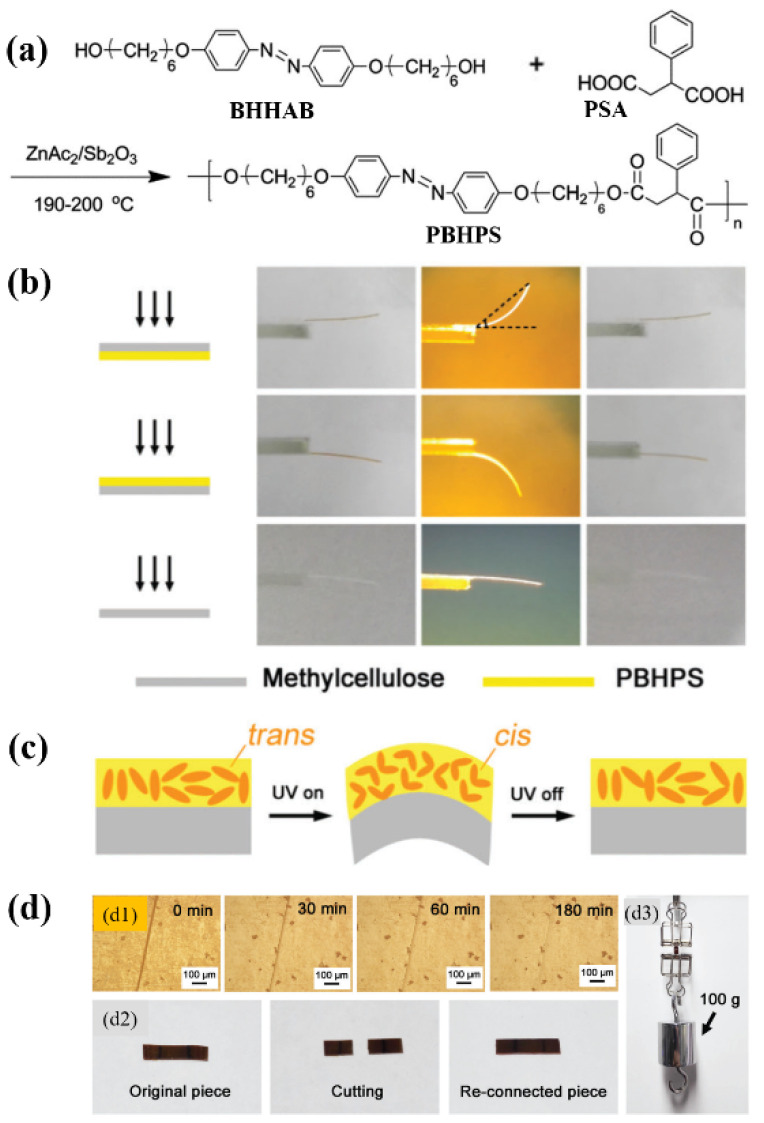
(**a**) Chemical structure and synthetic route of PBHPS. (**b**) Digital pictures of the P_0_._50_/MC_0_._50_ film motion with the UV light on or off (365 nm, 15 mW cm^−2^); (**c**) schematic illustration of light-driven deformation and shape recovery of the P/MC film; (**d1**–**d3**) self-healing process of PBHPS at 60 °C under the POM (**d1**), digital pictures of the reconnecting process for the broken pieces (**d2**), and the mechanical strength of the re-connected PBHPS piece with the loading of a 100 g weight (**d3**). Adapted with permission from Reference [50]. Copyright 2017 Royal Society of Chemistry.

π–π stacking interaction is a kind of weak interaction often existing between the aromatic rings, which has already been applied for the construction of supramolecular polymer systems [93]. To date, it has also been successfully used for developing physically crosslinked main-chain azo polymer photoactuators [50,51]. Wang and coworkers prepared a thermo- and photo-responsive main-chain azo LC polyester (namely PBHPS) via the polyesterification of 4,4′-bis(6-hydroxyhexyloxy)azobenzene (BHHAB) and 2-phenylsuccinic acid (PSA) (Figure 5a) [50]. PBHPS showed a relatively low *T*_g_ (26.9 °C by DSC) and a smectic LC phase. Strong π-π interaction was found to exist between its adjacent phenyl rings or between its side group and mesogenic unit. The π–π interaction-induced physical crosslinking imparted the unoriented PBHPS/methylcellulose (MC) bilayer film with reversible photoinduced bending behaviors (Figure 5b), which was attributed to the UV light-induced volume expansion of the PBHPS layer (Figure 5c). Moreover, it also endowed the PBHPS film with good shape memory and self-healing properties (Figure 5d). Later on, the same group also developed a series of novel LC copolyesters (namely P(BH-*co*-BP*n*)PS) with amphi-mesogenic units (including azo and biphenyl groups) via the one-pot melt polycondensation of BHHAB, 4,4′-bis(6-hydroxyhexyloxy)biphenyl (BHHBP), and PSA [51]. The resulting copolyesters showed good thermal stability, low *T*_g_ (around 25 °C by DSC), and smectic LC phases. The presence of the photoactive azo mesogen, photo-inert biphenyl group, LC phase, and π–π interaction (between the adjacent phenyl rings or between the side group and mesogenic unit) in these polymers allowed them to respond to external stimuli at the molecular level, leading to their thermal shape memory, reversible photoinduced bending/unbending, and self-healing behaviors. These, together with their reshaping and reprocessing ability owing to the physical crosslinking characteristics, make them highly promising materials for various smart devices.

### 2.4. Self-Assembly-Induced Physical Crosslinking

So far, some reprocessable azo polymer photoactuators have also been developed via the self-assembly-induced physical crosslinking, which mainly include those physically crosslinked ones based on the self-assembly of mesogens [52,53,54,55,56,57,58,59] and those based on the microphase separation of the azo block copolymers [60].

**Figure 6:**
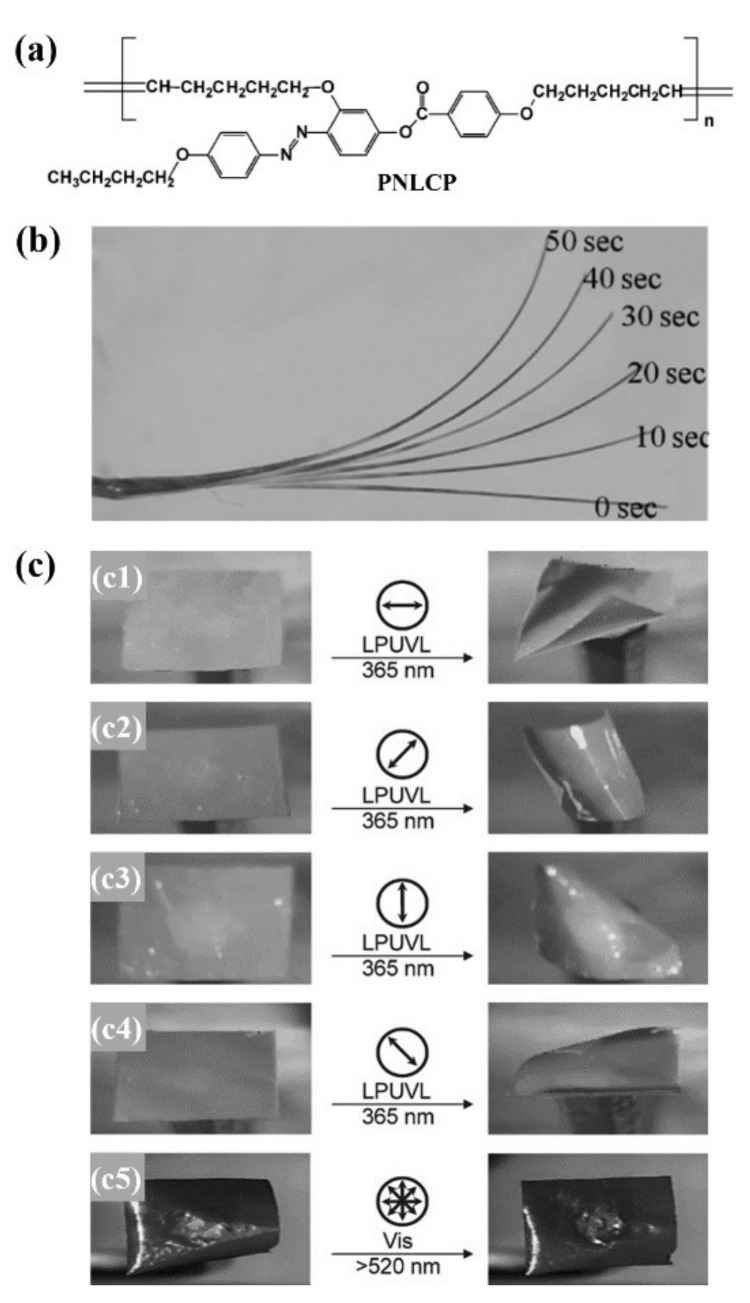
(**a**) Chemical structure of the photochromic LCP (PNLCP). (**b**) Superimposed photographic images of PNLCP fiber bending under UV irradiation. (**c**) Photographic images of precise control of bending direction using polarized light: (**c1**–**c4**) photomechanical bending of PNLCP films. Arrows in the circle indicate the direction of polarized light; (**c5**) photomechanical unbending induced by visible light (>520 nm). Reprinted with permission from Reference [52]. Copyright 2009 Royal Society of Chemistry.

Lee and Jeong’s group reported the fabrication of some photochromic 3D actuators from the uncrosslinked azo LCEs with self-assembled LC phase-induced physical crosslinking structures [52,53,54]. They prepared a main-chain polymer with azo mesogen at the flank via the acyclic diene metathesis polymerization (ADMET) of a novel photochromic LC monomer (PNLCM) (Figure 6a) [52,53]. The azo polymer had a *M*_w,GPC_ of 1.21 × 10^4^ g/mol, a *T*_g_ of 24 °C, and a nematic LC phase (*T*_cl_ = 145 °C). The uniaxially oriented azo polymer fiber fabricated via the melt spinning method bent toward the incident UV light (Figure 6b). The free-standing azo polymer film (of 20 μm thickness) prepared via the solution casting method also bent toward the light source upon exposure to both nonpolarized and polarized UV light. In the case of the nonpolarized UV light, the four corners of the film bent upward toward the light source at room temperature within 2 min owing to both the conformational transformation of polymer chains from the extended to the random-coil conformation (caused by *trans* to *cis* azo photoisomerization) and different degrees of the molecular orientational order change between the upper and lower layers of the film. In the case of the polarized UV light, only two sides of the film bent upward, and the bending direction had an approximately 45° angle to the direction of the polarized light (Figure 6c). This phenomenon was different from that observed by Ikeda and coworkers (their chemically crosslinked polydomain azo polymer film bent parallel to the direction of the polarized light [22]), which could be attributed to its special structure (i.e., the azo units have approximately a 42° angle with respect to the main-chain mesogenic units in the backbone). It was suggested that the microscopic domains having azo units parallel to the incident polarized light have the maximum response to the light-induced bending, thus resulting in a contraction of main chains in the direction approximately 45° to the incident polarized light. The weak intermolecular interactions between the mesogens ordered in the nematic LC phase were considered to act as the physical crosslinking sites and responsible for the photodeformation behaviors of this azo polymer.

In another work, Jeong and coworkers prepared a dendronized polymer (denpol, AZ_3_P) via the controlled ring-opening metathesis polymerization of an azo macromonomer AZ_3_M [54]. The azo denpol has a *M*_n,GPC_ of 173 kDa and three smectic LC phases (*T*_cl_ = 137 °C by DSC). A highly ordered free-standing AZ_3_P film (20 mm × 5 mm × 50 μm) with azo mesogen alignment homeotropic to the film surface was fabricated by using the melt scraping method. Upon exposure to the UV light, the AZ3P film bent away from the light source and resulted in the AZ_3_P film wrapping around the support pole. Irradiation of visible light could restore the AZ_3_P film to the initial floating form. Such photoinduced bending/unbending processes could be repeated many times without any noticeable degradation. A remotely controllable electric circuit was also built by using the actuating azo film with coated silver as the photoswitch.

**Figure 7:**
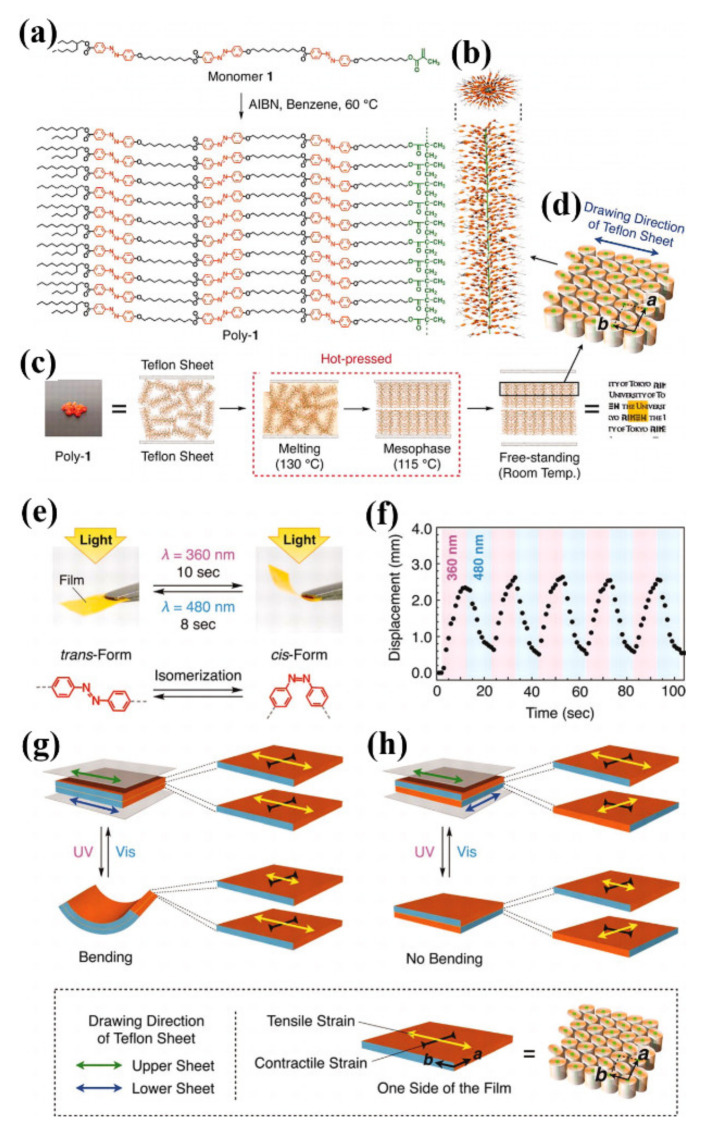
(**a**) Chemical structure and synthetic route of poly-1. (**b**) Schematic illustration of poly-1 adopting a cylindrical shape. (**c**) Schematic illustrations of the procedure for hot-pressing of poly-1 with uniaxially stretched Teflon sheets, together with those of plausible molecular events upon hotpressing. (**d**) Schematic illustration of the molecular arrangement in a hot-pressed film of poly-1. Blue arrow denotes the drawing direction of the Teflon sheet for hot-pressing. (**e**–**h**) Photomechanical responses of hot-pressed films of poly-1 prepared with parallel and orthogonally arranged Teflon sheets: (**e**) photographs of a hot-pressed film (5 mm × 6 mm × 10 μm) prepared with parallel-arranged Teflon sheets before (left) and after (right) exposure to UV light (λ = 360 ± 2 nm). The bent film recovered the initial flat shape upon exposure to visible light (λ = 480 ± 2 nm); (**f**) time-dependent change in displacement of the free edge of a hot-pressed film prepared with parallel-arranged Teflon sheets upon alternating irradiation with UV and visible lights; (**g**,**h**) schematic illustrations of tensile and contractile strains generated on one side of the films prepared with parallel (**g**) and orthogonally (**h**) arranged Teflon sheets. Reprinted with permission from Reference [55]. Copyright 2010 The American Association for the Advancement of Science.

In 2010, Aida and coworkers fabricated photodeformable free-standing films of large-area 3D molecular ordering from a comb-like polymer carrying three azo units in side chains (Poly-1) through one-step hot-processing with uniaxially stretched Teflon sheets (Figure 7a–c) [55]. Such films had the rectangular lattices oriented unidirectionally in the way that their *b* axis was parallel to the drawing direction of the Teflon sheets and the cylinders of Poly-1 aligned homeotropically with respect to the Teflon sheets used for hot pressing (Figure 7c,d). Moreover, a bimorph structure with a randomly oriented interior layer was sandwiched by a 2.3 μm thick unidirectionally oriented exterior layers on both sides. The poly-1 film (obtained through hot-processing with uniaxially stretched Teflon sheets of drawing directions parallel to one another) could exhibit reversible photoinduced rolling-up and flattening under the irradiation of UV and visible light alternately (Figure 7e,f). In contrast, the hot-pressed poly-1 film only showed limited photomobility when the drawing directions of the Teflon sheets (used for hot pressing) are orthogonal to one another. The different kinds of tensile and contractible strains generated on both sides of the films obtained with parallel- or orthogonal-arranged Teflon sheets were considered to be responsible for the different photodeformation behaviors of the above two kinds of films (Figure 7g,h). This one-step obtained 3D structural ordering of functional groups in a macroscopic length scale through combining a polymer brush as a scaffold with uniaxially stretched Teflon sheets might offer many new possibilities in the design of advanced functional materials.

By taking advantage of an azo polymer with LC lamellar structure-induced physical crosslinking, Yu and coworkers developed azo LCP microactuators that could manipulate fluid slugs by their photoinduced asymmetric deformation and the resulting capillary forces [56]. A strong and tough linear LCP with a long alkyl backbone containing isolated C=C double bonds and photoresponsive azo mesogens in side chains (*M*_n,GPC_ = 3.6 × 10^5^ g/mol, *M*_w_/*M*_n_ (or *Ð*) = 1.86) was prepared via the ring-opening metathesis polymerization. Free-standing structurally defined and robust tubular microactuators (TMAs) with arbitrary geometries were fabricated through first filling a glass capillary with an azo polymer solution in dichloromethane (~3 wt%), evaporating the solvent, annealing the coated capillary at 50 °C for 30 min, and then etching the glass capillary with hydrofluoric acid. The azo mesogens in the resulting TMAs self-assembled into a smectic phase with a zigzag tilting lamellar structure (with the tilt angle *ϕ* being 65° relative to the TMA film surface), which was considered to act as the physical crosslinking function. Upon exposure to an attenuated 470 nm light, the cross-sectional areas of the photodeformed TMAs at different irradiated positions increased with an increase in the light intensity, whereas the cross-sectional areas at different positions without irradiation remained almost the same. Therefore, the TMAs could deform to an asymmetric cone-like geometry, thus generating tunable capillary force to propel both simple liquids with a broad range of polarity and complex fluids in the direction of light attenuation. They thus hold great promise in many applications such as micro-pumps in microsystem technology and architecture without any aid from additional components. Later on, the same group also realized light-driven liquid manipulation in the flexible bilayer microtubes [57]. A newly designed azo LC polycyclooctene combining photoresponsive azo and photo-inert biphenyl moieties (with azo and biphenyl side chains at 1:2 ratio) (PABBP, *M*_n,GPC_ ≈ 3.0 × 10^5^ g mol^−1^, *Đ* ≈ 1.67) was synthesized via the ring-opening metathesis polymerization. A 1.5 m long bilayer photocontrollable flexible microtube (with a ≈ 100 μm thick ethylene-vinyl acetate (EVA) copolymer layer and a ≈ 25 μm thick PABBP layer) was obtained through coating the inner surface of a commercially available EVA copolymer microtube with a 5 wt% dichloromethane solution of PABBA, which also could propel various liquid slugs in the preset direction over a long distance due to the photodeformation-induced asymmetric capillary forces. Several light-driven prototypes of parallel array, closed-loop channel, multiple micropump, and a wearable device attached to a finger were also established by using the flexible bilayer microtubes to achieve liquid manipulation.

**Figure 8:**
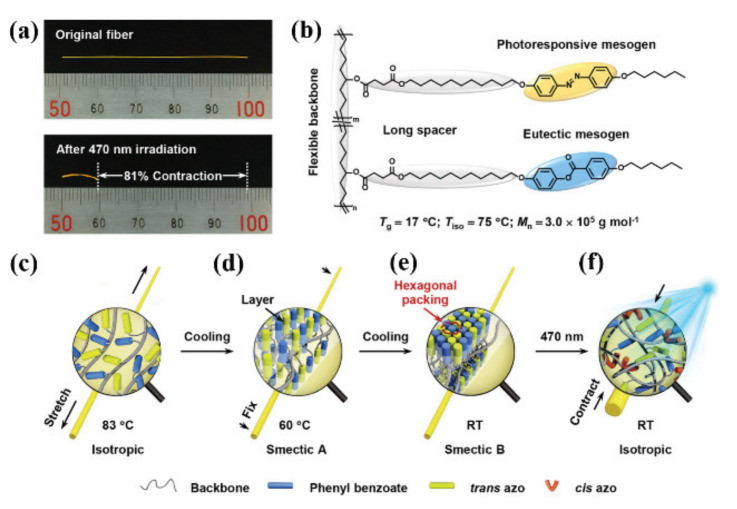
(**a**) Photographs showing light-driven contraction up to 81% of the azo LLCP fiber upon irradiation with 470 nm light (100 mW cm^−2^). (**b**) Molecular structure of a newly designed azo LLCP. *T*_g_, glass transition temperature; *T*_iso_, isotropic transition temperature; *M*_n_, number-average molecular weight. (**c**–**f**) Schematics showing the fabrication procedure of the azo LLCP fiber with highly ordered liquid crystal phase and mechanism of the light-driven ultralarge contraction based on *trans*–*cis* isomerization of azo mesogens. RT, room temperature. Reprinted with permission from Reference [58]. Copyright 2020 Wiley.

Reprocessable photodeformable azo polymer actuator with ultralarge contraction was also developed by Yu and coworkers (Figure 8) [58]. They developed a new strategy to realize a photoinduced ultralarge contraction of 81% for the azo linear LCP (LLCP) fibers by combining shape memory effect and photochemical phase transition (Figure 8a). An azo LC polycyclooctene with photoresponsive azo and photo-inert phenyl benzoate moieties (*M*_n,GPC_ = 3.0 × 10^5^ g mol^−1^, *Ð* = 1.62) was synthesized by ring-opening metathesis polymerization, which showed a *T*_g_ at 17 °C and was in a high elastic state at room temperature (Figure 8b). The fibers prepared via melt drawing had highly ordered smectic B (SmB) phase, which could constrain the movement of polymer backbones and lock the strain energy of the fibers (Figure 8c–e). Exposure of the fibers to 470 nm light at room temperature led to the reduction in *T*_g_ of the polymer and the movement of the polymer segments owing to the photoinduced *trans*–*cis*-*trans* cycles of the azo units, thus unlocking the strain energy stored in the fibers and leading to their ultralarge contraction (Figure 8f). These fibers were also used as the light-driven building blocks to achieve precise origami, to mimic the recovery of a “broken” spider web and to screen objects in different sizes.

Recently, Wu and coworkers fabricated a healable and reprocessable photoactuator with a new photodeformation mechanism (i.e., photoinduced solid-to-liquid transition) from the entangled side-chain azo LC polyacrylates prepared via atom transfer radical polymerization (ATRP) [59]. Flexible and stretchable free-standing films were fabricated from the entangled azo polymers with high molecular weights (*M*_n,GPC_ = 80–100 kg mol^−1^), which was impossible for the low-molecular-weight (5–53 kg mol^−1^) azo polymers without chain entanglements. The uniaxially oriented polymer film prepared via stretching at 90 °C (in the LC state) showed UV-induced bending (toward the light source) and visible light-induced unbending owing to the photoinduced changes in the order parameters on the irradiated side, just as the previous photodeformable azo polymers. The unstretched film without azo mesogen alignment also showed UV/visible light-induced bending/unbending (with their photomobile rates being slower than the stretched film), which proved to stem from the photoinduced solid-to-liquid transition of the azo polymers (the *trans*-azo polymer with *M*_n,GPC_ = 100 kg mol^−1^ had a *T*_g_ ≈ 80 °C, whereas the *cis*-rich same azo polymer obtained under UV illumination had a *T*_g_ ≈ 7 °C). Moreover, such azo polymer photoactuators could be healable and reprocessable via solution processing or light irradiation.

Hammond and coworkers demonstrated that a thermoplastic azo LC triblock copolymer with spherical polystyrene (PS) domains (as the physical crosslinking points) in a matrix of soft polysiloxane could be used as a rapid, room temperature photoactuator [60]. The azo triblock copolymer (i.e., PS-LCPVMS-PS: LCPVMS refers to LC poly(vinylmethylsiloxane) (PVMS) bearing pendant azo units, *M*_n,NMR_ = 111.6 kg/mol) was prepared through first the synthesis of a thermoplastic triblock copolymer elastomer with a functionalizable backbone (PS-PVMS-PS) via one-pot sequential anionic ring-opening polymerization and its subsequent attachment of side-on azo mesogens. It had two *T*_g_ (*T*_g_ = 15 °C for the LCPVMS block and around 100 °C for PS block) and a nematic LC phase between 15 and 100 °C. Upon turning on and off the UV light alternatively, the uniaxially oriented azo triblock copolymer film with the self-assembled physical crosslinking structures showed obvious and reversible extraction and extension under a tensional force supplied by the DMA instrument. In addition, the stress and strain occurred simultaneously, and the contraction corresponded to 4.22 μJ of work and an approximate maximum volumetric power of 0.458 mW/cm^3^.

## 3. Reprocessable Photodeformable Azo Polymers with Dynamic Covalent Bond (DCB)-Crosslinked Networks

Despite the significant progress made in the physically crosslinked photodeformable azo polymers, they typically have some disadvantages including their typical relatively lower thermal and anti-solvent capability. To address these issues, two types of reprocessable chemically crosslinked photodeformable azo polymers have been mainly developed by applying DCBs [61,62,63,64,65,66,67,68,69,70,71], which contain reversible chemical crosslinking [61,62,63] and rearrangeable (or exchangeable) chemical crosslinking [64,65,66,67,68,69,70,71], respectively. They have proven to show the advantages of both the chemically and physically crosslinked systems (i.e., high stability and good reprocessability).

DCBs are covalent bonds that can switch or exchange between two or several molecules under the appropriate external stimuli [108,109,110,111]. They have garnered tremendous interest in the field of polymer science because the incorporation of DCBs into polymers can endow them with many fascinating properties such as reprocessability, self-healability, shape-memory, high toughness, and various stimulus-responsivity. Following the milestone work by Leibler and coworkers in the preparation of crosslinked polymers with rearrangeable networks (or briefly “vitrimers”) through the transesterification between epoxy and ester bonds [112,113], many highly reprocessable crosslinked polymers have been developed by using DCB-forming reactions, including the reversible ones (e.g., redox-based thiol-disulfide switch and thermally reversible Diels–Alder (DA) reaction) and exchangeable ones (e.g., catalytically/thermally controlled transesterification, acid or base-catalyzed transthioesterfication, transimination reaction with or without catalysts, and thermo/photoinduced disulfide exchange) [108,109,110,111]. In the reversible pathway, the DCB linkages dissociate under certain stimulus, which can lead to a dramatic change in the chemical structures of the crosslinked polymers and their high recyclability. In the exchangeable pathway, the DCB linkages break and reform concurrently, which result in more or less constant crosslinking densities in the crosslinked polymers and thus their high reshaping ability (instead of recyclability). To date, some chemically crosslinked LC networks (LCNs) with reprocessability have also been prepared by using DCBs for different purposes [114,115,116]. In particular, recent years have witnessed significant progress made in the development of reprocessable photodeformable azo polymer actuators by using both reversible and exchangeable DCBs [61,62,63,64,65,66,67,68,69,70,71].

**Figure 9:**
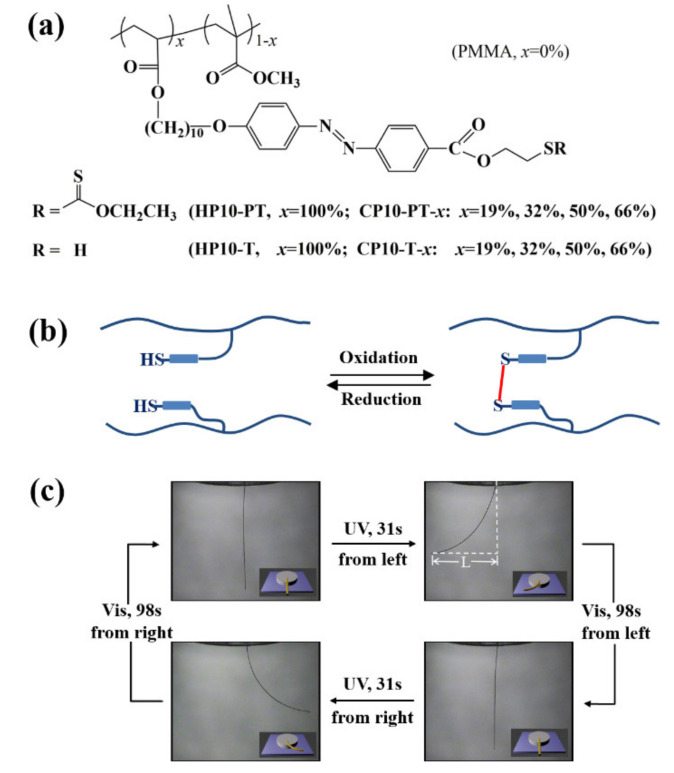
(**a**) Chemical structures of the side-chain polymers bearing azo mesogens with a protected thiol substituent (HP10-PT, CP10-PT-*x* (*x* refers to azo contents in the polymers)) (R = − (C = S)OCH_2_CH_3_) and those with free thiol-substituted azo mesogens (HP10-T, CP10-T-*x*) (R = H). (**b**) Schematic illustration of the reversible switches between thiol and disulfide groups in the uniaxially oriented azo polymer fibers via redox reactions. (**c**) Photographs of the post-crosslinked CP10-T-50% fiber that exhibits photoinduced bending and unbending upon irradiation with 365 nm UV light (150 mW cm^−2^) and visible light (>510 nm, 120 mW cm^−2^) at 40 °C. Reprinted with permission from Reference [61]. Copyright 2016 Royal Society of Chemistry.

In 2016, our group developed for the first time a facile and efficient approach for the preparation of recyclable photodeformable azo polymers with chemically crosslinked networks by using the reversible thiol-disulfide switches (Figure 9) [61]. A series of novel side-chain polymers bearing pendant thiol-substituted azo mesogens (i.e., HP10-T and CP10-T-*x*, Figure 9a) were prepared for such a purpose, which involved first the synthesis of side-chain polymers with protected thiol-substituted azo mesogens (HP10-PT, CP10-PT-*x*; *M*_n,GPC_ = 8550–16,300 g/mol, *Ð* = 1.11–1.45) via the free radical homopolymerization of an acrylate-type monomer bearing an azo mesogen with a protected thiol substituent or its copolymerization with methyl methacrylate and their subsequent treatment with *n*-butylamine to deprotect the thiol groups (Figure 9a). Uniaxially oriented fibers were readily fabricated from the side-chain copolymers bearing pendant thiol-substituted azo mesogens with relatively lower thiol contents (≤50%) via melt spinning method, whereas those with high thiol contents became quickly solidified when they were melted (probably caused by the occurrence of significant crosslinking owing to the oxidation of thiol groups into disulfide bonds). Following the post-crosslinking of the above fibers with H_2_O_2_ at room temperature (Figure 9b), uniaxially oriented fibers with disulfide bond-crosslinked networks were obtained. They showed rapid and reversible photoinduced bending and unbending under the irradiation of UV and visible light (Figure 9c) and good stability in terms of their resistance to higher temperatures and organic solvents. (For example, when immersed into a good solvent (e.g., chloroform) or heated to 50 °C, the uncrosslinked CP10-T-50% fiber quickly dissolved within 1 min or softened within 2 min, whereas its post-crosslinked fiber remained intact in the solvent or at 100 °C even after 12 h. In addition, the post-crosslinked fiber still showed excellent photoinduced bending and unbending after the solvent and heating treatment.) In particular, they could be readily recycled by using a reducing agent (tributylphosphine (TBP)) that can cleave the disulfide bonds into thiol groups (Figure 9b). More importantly, the recycled polymer (soluble in chloroform) could be used to reconstruct photodeformable fibers with disulfide-crosslinked networks, which showed reversible photoinduced bending and unbending rather similar to the initially prepared post-crosslinked fibers. The above strategy paves the way for efficiently fabricating various advanced azo polymer photoactuators of different physical formats (e.g., fiber, film, or other 3D shapes) with high mesogen alignment and excellent recycling and reconstruction ability after use owing to the reversible covalent characteristics.

Despite the versatility of our above strategy for the preparation of reprocessable photodeformable azo polymer actuators, the use of environmentally sensitive thiol groups makes the processability of the azo polymers troublesome. To address this issue, based on our previously reported post-crosslinking strategy [30,32], we have recently developed a new method for fabricating highly reprocessable, chemically crosslinked, photodeformable azo polymer actuators [62]. A series of environmentally stable side-chain polymers bearing *N*-hydroxysuccinimide (NHS) carboxylate-substituted azo mesogens were first prepared. Their uniaxially oriented fibers with reversible covalent networks were then obtained first by the fabrication of their uncrosslinked fibers via melt spinning method and their subsequent post-crosslinking with cystamine (a diamine containing a disulfide bond) under mild conditions. They not only showed rapid and reversible photoinduced bending/unbending at ambient temperature as well as high mechanical robustness and good solvent/heating stability, but also could be easily recycled into processable azo polymers in the presence of TBP. Particularly, since the post-crosslinking reaction only occurred on the thin surface layers of the azo polymer fibers, the recycled polymers in the first several (at least five) recycling processes still contained large amounts of NHS carboxylate-substituted azo units (together with a small amount of oxygen/heating-sensitive thiol-substituted ones). This characteristic allows highly efficient reconstruction of photodeformable fibers with excellent photomobility by applying melt spinning and post-crosslinking (with cystamine) methods. Note that all the post-crosslinked azo polymer fibers proved to be rather rigid and brittle, with their moduli ranging from 233 ± 9 to 555 ± 23 MPa and elongation at break being ≤ 8% under the stretching force. Moreover, the post-crosslinked fibers with moderately crosslinked networks showed higher elastic modulus than those with a lower crosslinked network. This strategy opens the new possibility to the facile and efficient development of various recyclable, advanced, chemically crosslinked photodriven actuators.

**Figure 10:**
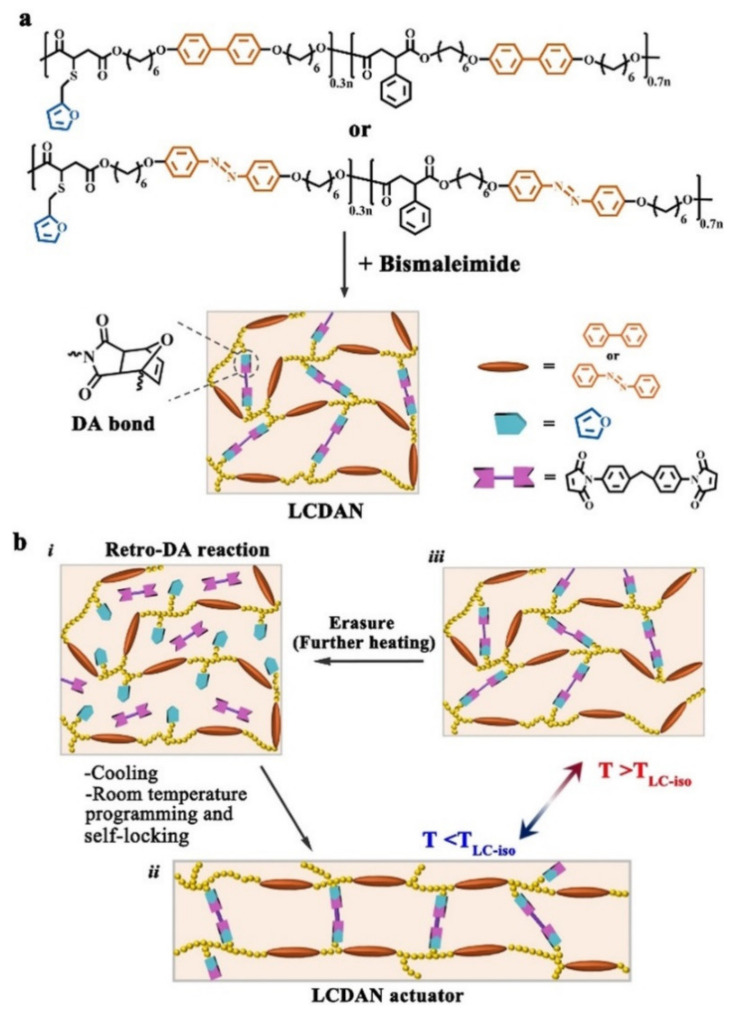
Design of the “self-lockable” LCDAN actuators. (**a**) Chemical structures of two main-chain LCPs bearing furan side groups and containing either biphenyl or azo mesogenic moieties, as well as the preparation of their LCDANs through DA-bonded crosslinking in the presence of bismaleimide. (**b**) A schematic showing the room temperature programming and self-locking of LCDAN actuators capable of reversible shape change upon the LC isotropic (order–disorder) phase transition. Reprinted with permission from Reference [63]. Copyright 2020 Wiley.

Zhao and coworkers also reported recyclable and reprocessable chemically crosslinked photodeformable azo polymer actuators (with room temperature programmability and solution reprocessability) based on LC DA dynamic networks (LCDANs) (Figure 10) [63]. Two main-chain LC polyesters bearing furan side groups and either biphenyl or azo mesogenic units in the backbones (i.e., BP-LCP and AZO-LCP) were synthesized and used to form LCDANs with a bismaleimide compound (Figure 10a). By taking advantage of the thermally reversible nature of the furan-maleimide DA reaction (especially the rather slow reforming nature of the DA bond at room temperature), the resulting LCDANs can be readily reshaped or programmed into various 3D-structured actuators (such as strips, helices, origami, and different 3D solid shapes) through first triggering the reverse DA reaction (i.e., DA bond dissociation) by heating them to 125 °C and then cooling them to room temperature for 3D shape reprogramming. Most importantly, they can be self-locked by the slowly formed DA bonds for reversible shape transformation without requiring additional thermally or optically initiated chain crosslinking reaction (Figure 10b). Moreover, some actuators that exhibited UV light-driven caterpillar-like crawling and wheel rotating locomotion were also fabricated by laminating an oriented AZO-LCDAN strip with a polyimide film (Kapton). In particular, the LCDANs could be easily reprocessed by dissolving them into solutions at 120 °C or heating them into melts (at >155 °C), which are impossible for the photodeformable LCN vitrimers with typical DCBs.

**Figure 11:**
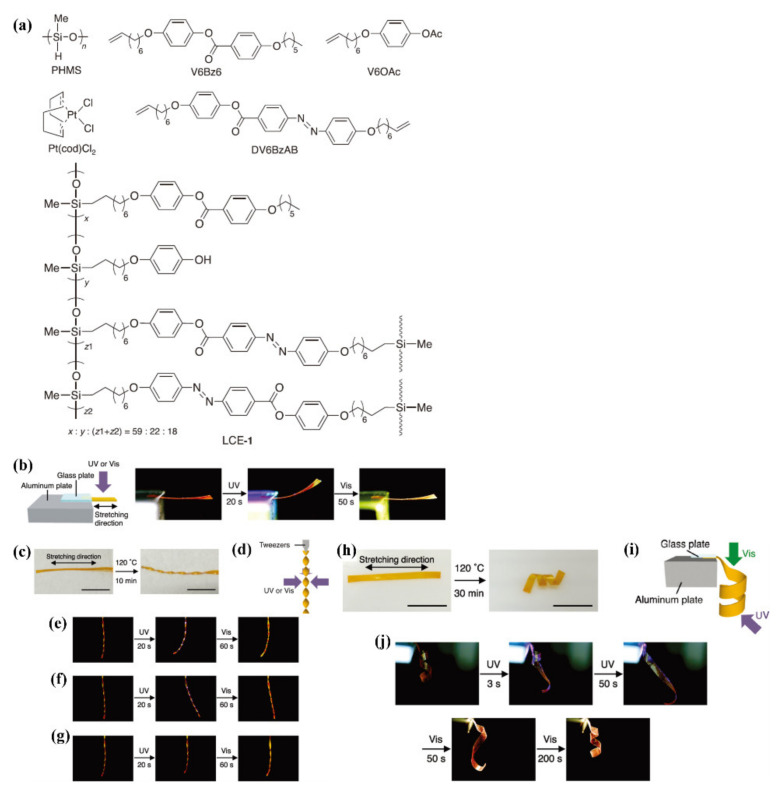
(**a**) Chemical structures of the compounds used to prepare LCE and LCE with dynamic covalent bonds. (**b**) Photoinduced bending of a monodomain LCE-1 film upon irradiation with UV (365 nm, 35 mW cm^−2^) and visible light (>540 nm, 67 mW cm^−2^) at room temperature. Size of the film: 7 mm × 2 mm × 60 μm. (**c**–**j**) Photoinduced deformation of reshaped LCE films: (**c**) reshaping of a monodomain LCE film into a helicoid. Scale bars: 5 mm. Size of the film before reshaping: 14 mm × 0.8 mm × 80 μm; (**d**) experimental setup for the observation of photoinduced deformation of the helicoid; (**e**–**g**) photoinduced deformation of the helicoid upon irradiation with UV (365 nm, 120 mW cm^−2^) and visible light (>540 nm, 80 mW cm^−2^) at room temperature from the left (**e**), the right (**f**), and back (**g**); (**h**) reshaping of a monodomain LCE film into a spiral ribbon. Scale bars: 10 mm. Size of the film before reshaping: 25 mm × 2 mm × 70 μm; (**i**) experimental setup for the observation of photoinduced deformation of the spiral ribbon; (**j**) photoinduced deformation of the spiral ribbon upon irradiation with UV (365 nm, 97 mW cm^−2^) and visible light (>540 nm, 60 mW cm^−2^) at room temperature. Reprinted with permission from Reference [65]. Copyright 2016 Wiley.

Recently, some photodeformable azo polymers with rearrangeable covalent networks have also been developed by using the exchangeable DCBs, which could also show high reprocessability (or reshaping capability) (but they typically cannot be recycled) [64,65,66,67,68,69,70,71]. Ikeda’s group pioneered this research direction [64,65,66,67]. For example, they prepared a polysiloxane-type LCE by reacting poly (hydrogenmethylsiloxane) (PHMS) with some vinyl compounds including V6Bz6, V6OAc, and DV6BzAB in the presence of a Pt catalyst (Figure 11a) [65]. The resulting product (LCE-1) contains crosslinks with both azo and ester groups and hydroxylated side chains. The occurrence of the transesterification between ester crosslinks and hydroxyl groups led to the exchange of links and thus the rearrangement of the network topology, while maintaining the number of the crosslinks constant during the reaction. By utilizing this rearrangeable covalent network, chemically crosslinked azo polymer films with a high alignment order of mesogens and reversible photoinduced bending/unbending behaviors were fabricated by raising the temperature of the randomly oriented crosslinked film in an elongated state to 120 °C (in the LC state) for a certain time (Figure 11b). Moreover, the flat monodomain films of LCE-1 were also transformed into complicated 3D shapes such as a helicoid and a spiral ribbon under strain at 120 °C, which exhibited photoinduced bending toward various directions and winding/unwinding motions, respectively (Figure 11c–j).

**Figure 12:**
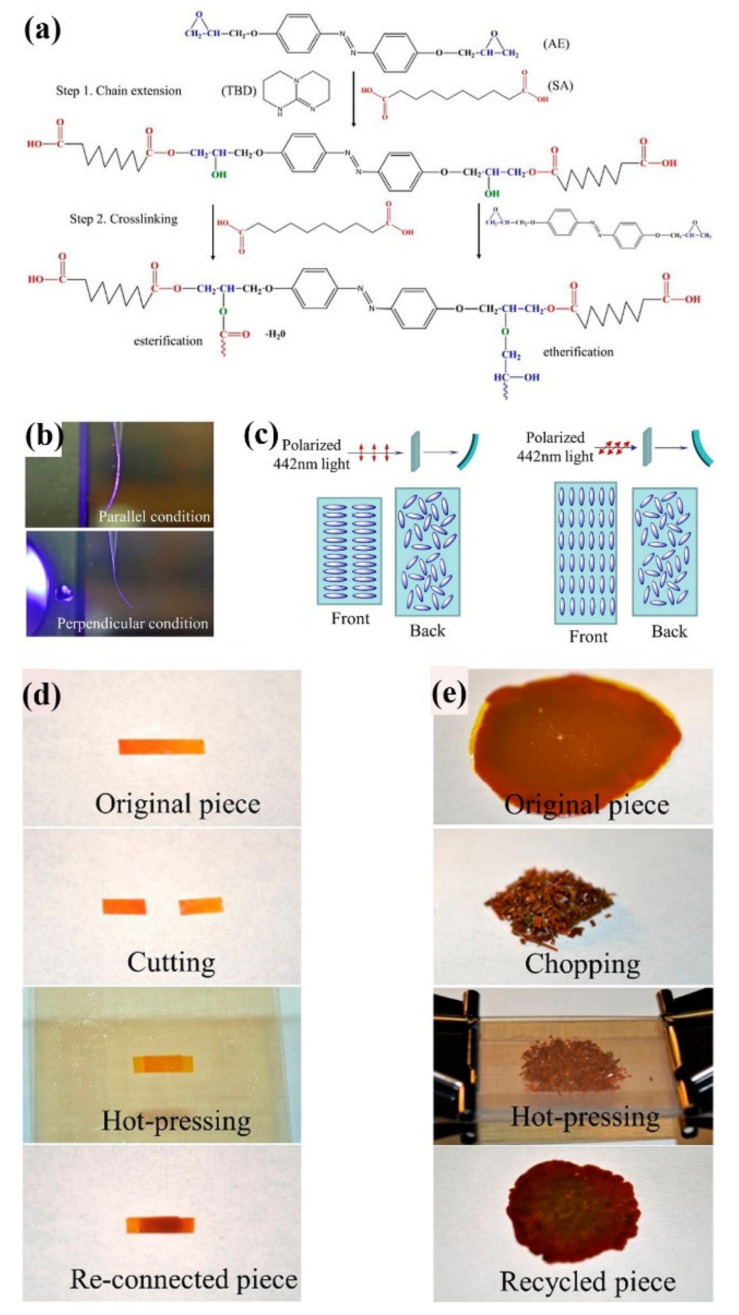
(**a**) Synthesis route and reaction mechanism of multifunctional LCNs. (**b**,**c**) Photomechanical behavior of LCNs: (**b**) blue light-induced deformation of LCN film (10 mm × 1 mm × 15 μm) at a light intensity of 40 mW/cm^2^; top: light polarization direction parallel to the long axis of the LCN film; bottom: light polarization direction perpendicular to the long axis of the LCN film; (**c**) mechanism of bidirectional bending behaviors of the LCNs under blue light irradiation. (**d**,**e**) Reprocessability of the LCNs: (**d**) reconnecting broken pieces using a transesterification reaction; (**e**) reprocessing LCN pieces by hot pressing. Reprinted with permission from Reference [68]. Copyright 2016 American Chemical Society.

Kessler and coworkers prepared reprocessable photoresponsive LC epoxy networks with shape memory and photodeformation behaviors by incorporating dynamic ester covalent bonds (Figure 12) [68]. They first synthesized a multifunctional LCN bearing three different functional building blocks (i.e., azo chromophore, LC, and dynamic ester bond) by reacting an azo epoxy monomer (4,4′-diglycidyloxyazobenzene, AE) and a dicarboxylic acid curing agent (sebacic acid, SA) in the presence of a ring-opening/transesterification catalyst (TBD) (Figure 12a). A polydomain LCN film was prepared by first curing a mixture of AE, SA, and TBD at 170 °C for 1 h in the parallel plate fixture of a strain-controlled rheometer and its subsequent curing at 200 °C for 3 h in a convection oven. It showed obvious photoinduced bending upon exposure to the polarized 442 nm light (Figure 12b,c). In addition, it also exhibited triple shape memory behavior and good UV-induced self-healing properties (Figure 12d). In particular, excellent reprocessability was also achieved for such a LCN owing to its efficient transesterification reaction induced by the dynamic ester bond at appropriate temperatures (Figure 12e). Later on, the same group also fabricated reprocessable photoresponsive LC epoxy networks with exchangeable disulfide bonds by simply adding a disulfide bond-containing dicarboxylic acid curing agent into the above system and replacing the dual function (i.e., ring-opening and transesterification) catalyst TBD with an only ring-opening catalyst (imidazole) [69]. In addition to showing various photomechanical and shape memory properties, the resulting LCNs could also be reshaped, repaired, and recycled owing to their incorporation of the dynamic disulfide bonds that could take place in both disulfide–disulfide exchange and thiol–disulfide interchange reactions.

Reprocessable photodeformable azo polymer actuators were also developed by Xia and coworkers through introducing dynamic ester bonds into the crosslinked azo polymers [70,71]. Following a similar synthetic strategy as in Kessler’s work [68], they prepared the malleable chemically crosslinked LCP network through the reaction between 4,4′-diglycidyloxyazobenzene and a dicarboxylic acid (i.e., dodecanedioic acid) (1:1) in the presence of TBD [70]. The resulting polymer had a *T*_g_ of around 55 °C and a smectic-isotropic phase transition (*T*_si_) at about 122 °C. Its stretched thin films with different alignment degrees of azo mesogens and polymer chains were fabricated by first preparing the unstretched film through hot-compressing the polymer granules at 180 °C and cooling to room temperature and subsequently stretching the obtained film to different extents at 100 °C. The combined effect of the azo *trans*–*cis* photoisomerization and the strain energy stored in highly extended polymer chains led to the largely amplified photoinduced contraction force (up to about 7 MPa), which could also be easily tuned by adjusting the film strain degree. Some light-driven moving 3D objects with robust, tunable, and continuous motions at the macroscopic scale, such as wheels and spring-like ribbons, were also fabricated by using the stretched ALCE films laminated to a transparent biaxially oriented polypropylene film. Later on, they further developed a reprocessable dual (i.e., NIR− and UV light−) responsive polymer nanocomposite with good processability and enhanced photocontrol of actuations by doping polymer-grafted gold nanorods (AuNRs, inducing photothermal effect) into the above dynamic azo LCNs [71]. Different formats of actuators with photocontrolled motions were fabricated, including the stretched films that showed reversible photoinduced bending/unbending, plastic “athletes” that executed light-controlled push-ups and sit-ups, a light-driven caterpillar-inspired walker that crawled forward on a ratcheted substrate, and a polymer “crane” that could perform light-controlled sophisticated, concerted robot-like, and macroscopic motions.

## 4. Some Other Reprocessable Uncrosslinked Photodeformable Azo Polymers

In addition to the above-described reprocessable physically and DCB-crosslinked photodeformable azo polymers, some special reprocessable photodeformable azo polymers without the above dynamic crosslinking networks (briefly uncrosslinked photodeformable azo polymers) have also been reported, which mainly include linear azo polyimides developed by White and coworkers [72,73,74,75,76,77] and amphiphilic epoxy-based azo random copolymers developed by Wang and coworkers [78,79,80,81,82,83,84,85,86].

**Figure 13:**
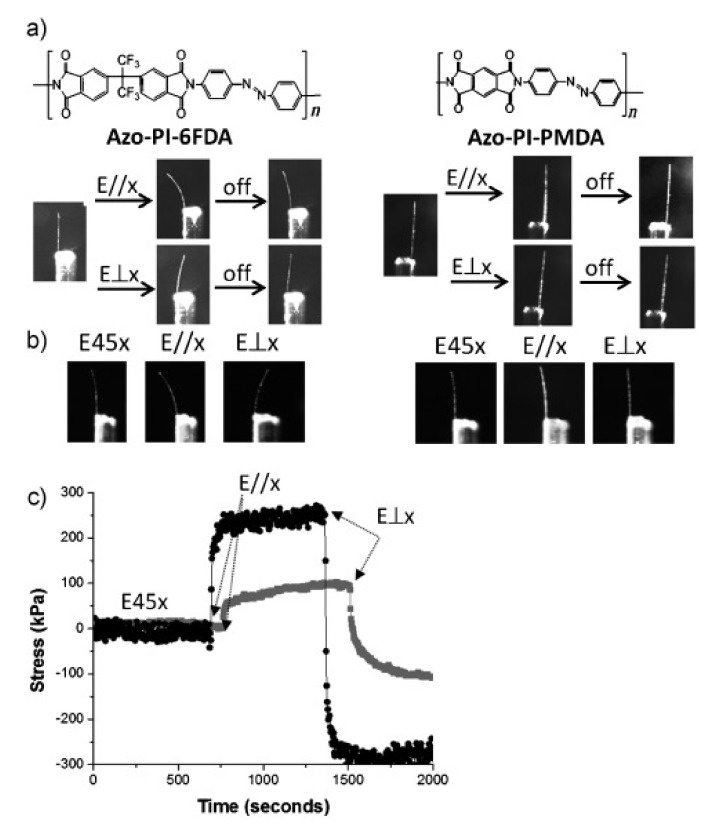
Polarization−dependent photomechanical response of Azo-PI-6FDA upon irradiation with 100 mW cm^−2^ of *λ* = 442 nm light (**a**) and Azo-PI-PMDA upon irradiation with 100 mW cm^−2^ of *λ* = 488 nm (**b**). The light was polarized parallel to the long axis (E//x) of the 5 mm × 0.5 mm × 0.02 mm cantilever and orthogonal to the long axis of the cantilever (E⊥x). (**c**) The tensile stress generated from Azo-PI-6FDA (●) and Azo-PI-PMDA (■) upon irradiation with 100 mW cm^−2^ of *λ* = 457, 488, and 514 nm multiline output from an Argon ion laser is examined during continuous irradiation to linearly polarized light oriented 45°, parallel, and orthogonal to the long axis of the 6 mm × 1 mm × 0.02 mm gauge. Reprinted with permission from Reference [72]. Copyright 2012 Wiley.

In 2012, White and coworkers reported the photomechanical deformation of azo-functionalized polyimides (Figure 13) [72]. A series of amorphous or semi-crystalline linear main-chain azo polyimides were prepared via the condensation polymerization of 4,4′-diaminoazobenzene with different dianhydrides (Figure 13a). They have *T*_g_ values ranging from 360 to >450 °C and storage moduli (*E*’) in the range of 3.8–6.2 GPa. Upon exposure to the polarized light (λ = 442 and 488 nm), the amorphous azo polyimide (Azo-PI-6FDA) cantilevers could show obvious bending toward and away from the incident light when the direction of the polarized light was parallel and perpendicular to the long axis of the cantilevers, respectively (Figure 13b, left). A photogenerated tensile stress of up to 265 kPa was generated in these materials (λ = 442 nm, 100 mW cm^−2^) (Figure 13c). In contrast, the semi-crystalline azo polyimide (Azo-PI-PMDA) samples hardly showed photomobility under the same photoirradiation condition (Figure 13b, right). A general rule was observed that increasing crystallinity reduces the magnitude of bending and tensile stress, which could be attributed to the reduction in the photoresponsivity of the polymer systems with crystalline contents.

Later on, the same group further prepared a series of main-chain azo polyimides with different chemical structures and studied the effects of the backbone rigidity [74], segmental mobility [75], free volumes [76], and crosslinking [75,77] on the photomechanical bending and relaxation of these samples [77]. The following rules were obtained: (1) increasing the rigidity of the polymer backbone can lead to the increased magnitude of the generated stress but decreased bending angles of the cantilevers [74]; (2) the inclusion of a bulky cardo substituent into the azo polyimide can strongly increase its fractional free volume, which is conducive to the more efficient photoisomerization or reorientation of azo units and thus leads to comparatively larger photoinduced deformation and force generation [76]; (3) azo polyimide materials with larger segmental mobility can assimilate larger force generation and displacement; (4) crosslinking the rigid backbone polymer provides a network environment containing additional free volume, which is coupled with network connectivity of the crosslinked chains, leads to enhanced light-induced deformation [75,77].

**Figure 14:**
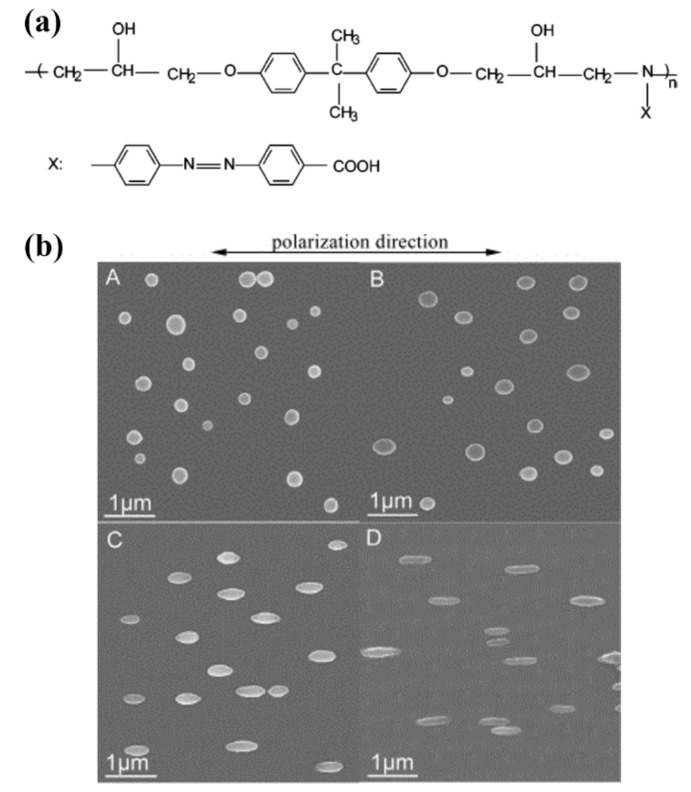
(**a**) Chemical structure of the polymer (BP-AZ-CA); (**b**) SEM images of colloidal spheres before irradiation (**A**) and after being exposed to interfering polarized Ar+ laser beams for different times: (**B**) 5 min, *l*/*d* = 1.31; (**C**) 12 min, *l*/*d* = 2.03; (**D**) 15 min, *l*/*d* = 2.35. Reprinted with permission from Reference [78]. Copyright 2005 American Chemical Society.

A number of photodeformable colloid particles fabricated by the self-assembly of amphiphilic azo polymers have also been developed [78,79,80,81,82,83,84,85,86]. Wang’s group first discovered this unique photoinduced shape deformation of colloid particles that were prepared by the self-assembly of an amphiphilic epoxy-based random copolymer bearing push-pull-type azo units (Figure 14a,b) [78]. Such spherical colloid particles suspending in the solutions changed from a spherical shape into an ellipsoid under the irradiation of interfering linearly polarized laser beams (Figure 14b). The elongation direction of the particles was parallel to the polarization direction of the laser beam. In addition, the deformation degrees of the colloid particles were enhanced by increasing the light intensity [79] and the azo densities inside the colloid particles [80] and by choosing appropriate colloid particle sizes [81] and substituent groups [82]. Later on, they also demonstrated the similar photodeformation behaviors for the colloid particles prepared via the self-assembly of azo block copolymers [83,84,85]. The photomechanical force generated by the photoisomerization of azo units inside the colloid particles was believed to lead to their photoinduced mass migration and thus shape deformation [86].

## 5. Conclusions and Outlook

The above detailed overview of the reprocessable photodeformable azo polymers clearly demonstrates that significant advances have been made in this burgeoning area. So far, a large number of photodeformable azo polymers with outstanding reprocessability and high actuating performance have been developed, and many different synthetic strategies to obtaining such photodeformable polymers are available. Of special note is the physically crosslinked photodeformable main-chain azo polymers, which represent one type of advanced reprocessable photodeformable materials highly promising in many photoactuating applications because of their typically high mechanical robustness and physical stability, excellent recyclability and reprocessability, and superior photomechanical effects. Moreover, the fully recyclable photodeformable azo polymers with reversible DCB-crosslinked networks that can be reprocessed at room temperature also hold much promise. In particular, some breakthroughs have been achieved in preparing reprocessable photodeformable azo polymers with ultralarge contraction [58], strong photoinduced stress (up to 7 MPa) [66], and various complex 3D motions [1,2,3,4,5,6,7,8,9,10,11,12,13,14,15].

Despite the remarkable success achieved in this area, the following issues still need to be addressed to promote the development of more advanced reprocessable photodeformable azo polymer actuators. First, although physically crosslinked photodeformable main-chain azo polymers and those with DCB-crosslinked networks show great application potential, they are still rather limited. Further efforts should be devoted to developing more versatile synthetic strategies for obtaining such photodeformable azo polymers with high mechanical and photomechanical properties (e.g., those with good film-forming and room temperature photoactuating ability). Second, a deep understanding of the structure–property relationship of many such photomechanical systems (for instance, the effects of the types of H-bonding-forming groups and their location and densities in the main-chain azo polymers as well as the molecular weights on their mechanical and photomechanical properties) is still missing. More studies in this direction are definitely desirable because it is of paramount importance for rationally designing more advanced reprocessable photodeformable azo polymers. Third, to date, the studies on the presently developed reprocessable photodeformable polymers have been mainly focused on their photomechanical effects (e.g., photomobile rates, 3D motions, actuating mechanism, etc.) and reprocessability. Those with multifunctional or multiresponsive properties are highly appealing for different application scenarios and should thus be the focus of future work.

Although this research field is still in its infancy, we are optimistic for the bright future of these advanced functional polymers. We expect that more significant progress will take place with the combined efforts from scientists working in the fields of polymer chemistry, polymer physics, materials science, photochemistry, and engineering technology and it will eventually revolutionize the whole field of photoactuators.
